# Involving families in psychiatric treatment and rehabilitation

**DOI:** 10.1192/j.eurpsy.2021.418

**Published:** 2021-08-13

**Authors:** C. Fernandes Santos, A.B. Medeiros, R. Gomes

**Affiliations:** Psychiatry And Mental Health Department, Hospital Garcia de Orta, E.P.E., Almada, Portugal, Portugal

**Keywords:** family interventions, psychiatry, recovery, Rehabilitation

## Abstract

**Introduction:**

Psychiatric rehabilitation promotes recovery in individuals with mental disabilities. Its mission is to engage patients and families or caregivers in a collaborative treatment process. The vision of recovery is more likely to become a reality when patients and families are actively involved in treatment. Numerous factors have converged during the past decades to facilitate development and refinement of evidence-based approaches for strengthening families coping with mental disorders.

**Objectives:**

To review current knowledge on the importance of involving families in psychiatric treatment and rehabilitation, addressing effectiveness of family interventions, role of family coping skills in neutralizing stress and vulnerability, and family burden of mental illness.

**Methods:**

Non-systematic review of literature through search on PubMed/MEDLINE database for publications up to 2020. Textbooks were consulted.

**Results:**

Given the unpredictability of major mental disorders, families assume responsibility for extensive monitoring and supervision of a severely and chronically mentally ill relative. Clinical, social, family and economic benefits are achieved by adding psychosocial family interventions to a comprehensive array of services required by patients. Family interventions are not stand-alone modalities: they are coordinated with pharmacotherapy, illness management, crisis intervention, clinical case management, skills training and supportive services. Family interventions show benefits, such as fewer psychotic/affective episodes of exacerbation or relapse by the patient, reduced hospitalizations and improved family morale and less emotional burden.

**Conclusions:**

The new and effective family interventions do not stigmatize families as being ‘sick’ or in need of therapy to ‘straighten them out’. Family interventions are viewed as conferring added therapeutic protection to the patient and relatives.

**Disclosure:**

No significant relationships.

